# Bridging the gap

**DOI:** 10.7554/eLife.19733

**Published:** 2016-08-23

**Authors:** Sarah K Armitage, Sergey V Plotnikov

**Affiliations:** Department of Cell and Systems Biology, University of Toronto, Toronto, Canada; Department of Cell and Systems Biology, University of Toronto, Toronto, Canadasergey.plotnikov@utoronto.ca

**Keywords:** microtubule, focal adhesion, talin, Human

## Abstract

A new study reveals that a protein called talin forms a vital link between microtubules and focal adhesions at the surface of cells.

**Related research article** Bouchet BP, Gough RE, Ammon Y-C, van de Willige D, Post H, Jacquemet G, Altelaar AFM, Heck AJR, Goult BT, Akhmanova A. 2016. Talin-KANK1 interaction controls the recruitment of cortical microtubule stabilizing complexes to focal adhesions. *eLife* **5**:e18124. doi: 10.7554/eLife.18124

**Image** The adaptor protein KANK1 (green) clusters around focal adhesions (red)

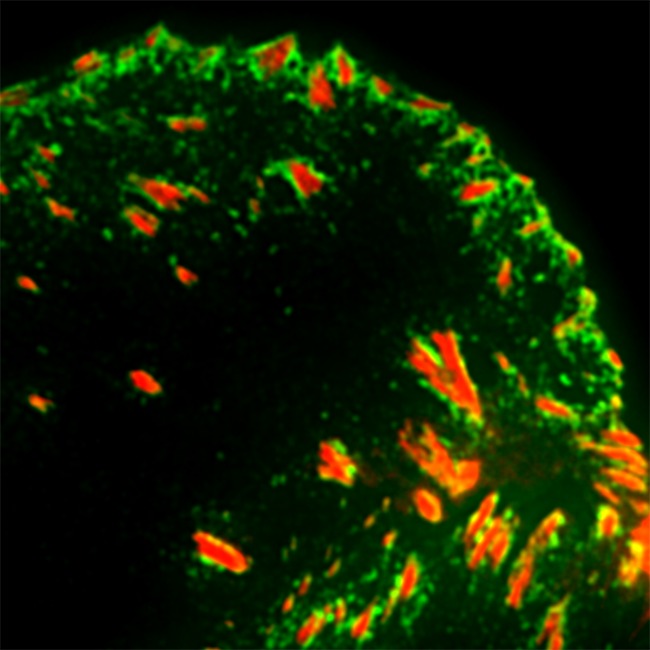

The extracellular matrix is a scaffold-like framework that surrounds and supports the cells in an animal’s tissues and organs. The cells attach to this matrix via structures on the cell surface known as focal adhesions; these structures contain a number of proteins including integrins.

Integrins span the surface membrane of the cell and essentially connect the extracellular matrix with the cell’s internal support structure: the cytoskeleton. Changing the interactions between focal adhesions and the underlying cytoskeleton is crucial for a variety of processes in living cells (Stehbens and Wittmann, 2012; Etienne-Manneville, 2013). This dynamic interplay relies on the ability of the cytoskeleton to bind temporarily to the focal adhesions in a highly regulated manner. Now, in eLife, Anna Akhmanova, Benjamin Goult and colleagues at Utrecht University, University of Kent and University of Turku unravel some of the molecular details behind these interactions (Bouchet et al., 2016).

The cytoskeleton of an animal cell is a complex network of interlinking filaments, including microfilaments and hollow cylinders called microtubules that extend throughout most of the cell. Microfilaments are made from a protein called actin, and the actin cytoskeleton plays a major role in focal adhesion assembly and maturation (reviewed in Gardel et al., 2010).

However, research conducted over a decade ago implicated microtubules as potent regulators of focal adhesions in migrating cells. For a cell to move within a tissue, its existing focal adhesions must be broken down. Microtubules were shown to repeatedly target growing focal adhesions, halting growth and initiating their disassembly and cause them to stop growing and disassemble instead (Kaverina et al., 1998; 1999). This occurred across the entire cell, but was more common at the cell’s rear. These findings suggested that the targeting events may efficiently control how focal adhesions behave across the entire cell (Kaverina and Straube, 2011).

How microtubules get to the focal adhesions, however, has been a long-standing question. It is plausible that microtubules grow along actin filaments towards focal adhesions (Etienne-Manneville, 2013). Another possible mechanism could be stabilization of microtubule tips at the cell surface membrane. In fact, protein complexes that contain several membrane-associated proteins and proteins that bind to the end of microtubules are believed to target microtubules to focal adhesions (Lim et al., 2016; Lansbergen et al., 2006; Stehbens et al., 2014). These multi-subunit structures are termed CMSCs (short for cortical microtubule stabilization complexes), but the exact linker of CMSCs to focal adhesions was previously unknown.

Akhmanova, Goult and colleagues, including Benjamin Bouchet as first author, have now identified two major players that bridge microtubules to the focal adhesions (Bouchet et al., 2016). First, Bouchet et al. demonstrated that the CMSC can only assemble if the adhesion site is under mechanical tension. Indeed, small molecule drugs that suppress the molecular motors that generate this tension also prevent the assembly of CMSCs. This finding led Bouchet et al. to ask whether force-bearing molecules in the focal adhesions were directly involved in assembling and localizing the CMSCs. The answer to this question was yes: talin, a core component of focal adhesions, can link to microtubules by binding to an adaptor protein in the CMSC called KANK1 (Figure 1A).

**Figure 1.**
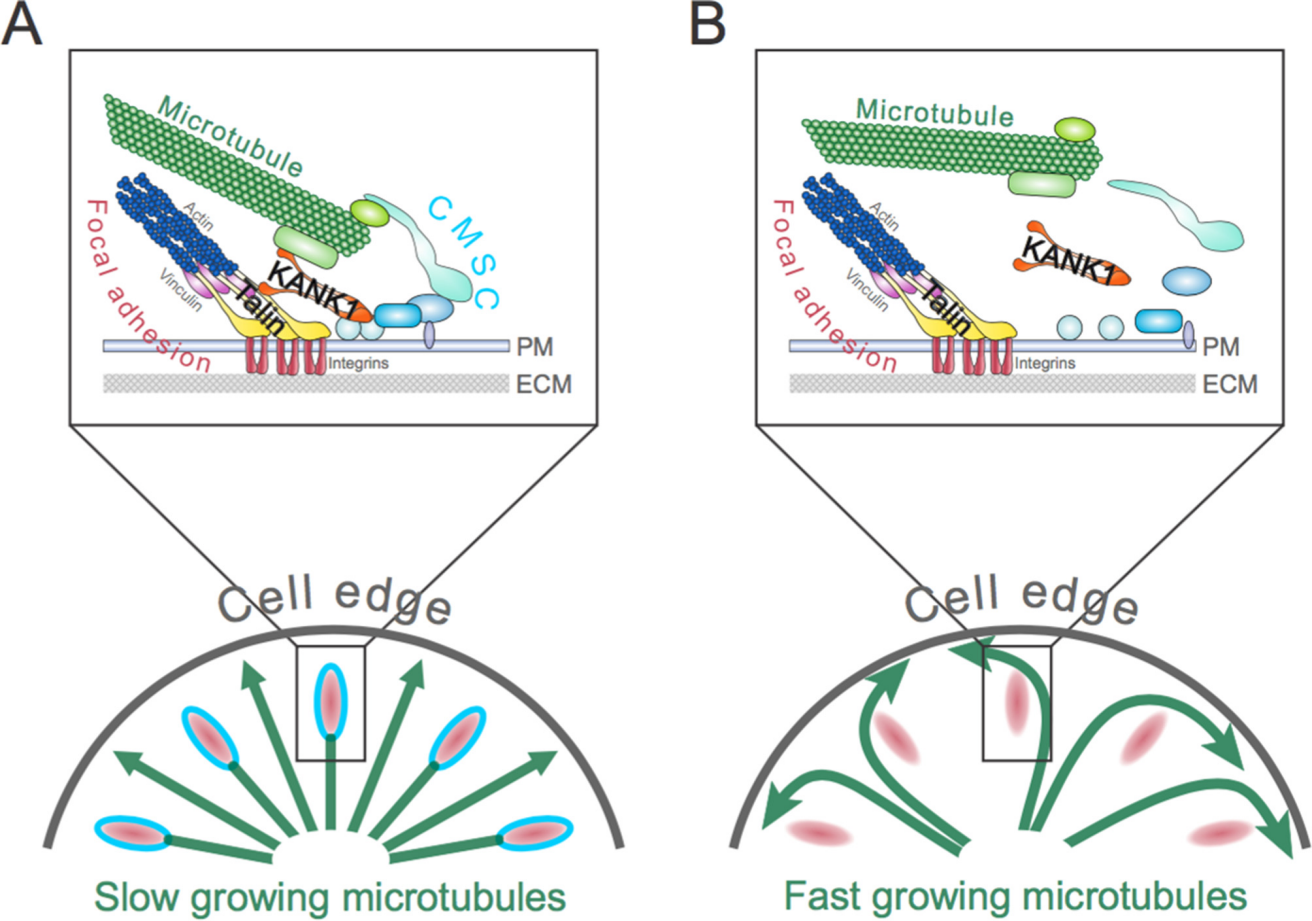
The role of the talin-KANK1 interaction in controlling the architecture of microtubules. (**A**) The cortical microtubule stabilization complex (CMSC) is a collection of proteins that associates with the plasma membrane (PM) of human cells (top panel). Individual components of the complex are shown in different shades of blue, while microtubule end-binding proteins are shown in light green. Focal adhesions are multi-protein assemblies containing integrins (shown in red) that connect the cell to the surrounding extracellular matrix (ECM). Talin and vinculin (shown in yellow and purple, respectively) link integrins to the actin cytoskeleton. Bouchet et al. report that a mechanically sensitive adaptor protein talin must interact with another protein, called KANK1 (orange), to link the CMSC to focal adhesions. This interaction prevents microtubules from growing too much at the edge of the cell (bottom panel). The green arrows depict growing microtubules, and the speed of microtubule growth is depicted by the size of the arrowhead. (**B**) If the talin-KANK1 interaction is disrupted, CMSC components are not recruited to focal adhesions and the CMSC cannot capture microtubules (top panel). Disrupting the talin-KANK1 interaction does not, however, stop focal adhesions from assembling. Disrupting the talin-KANK1 interaction leads to the microtubules growing faster and becoming disorganized at the cell’s edge (bottom panel).

Several domains of the talin protein can stretch and relax to buffer the mechanical tension applied to the molecule. This means that mechanical forces can alter how talin interacts with other proteins (del Rio et al., 2009). Bouchet et al. mapped exactly where KANK1 binds to talin, and found that KANK1 binds to a part of the talin molecule called the rod domain. Applied tension causes the rod domain to unfold, exposing a hidden site that can bind the actin-associated protein vinculin, and reinforcing the actin anchoring (Yao et al., 2016).

Mutation analysis then confirmed that talin and KANK1 interact in cells, allowing Bouchet et al. to identify mutant versions of the proteins that do not interact with each other. These disruptive mutations had a dramatic effect on the organization of the CMSC and microtubule dynamics in human cells grown in the laboratory (Figure 1B). Replacing the cells’ own talin with the mutant that could not bind to KANK1 excluded KANK1 from the adhesion sites and also prevented the other CMSC components from accumulating at focal adhesions. Furthermore, the disruption of the CMSCs coincided with the microtubules becoming less organized – particularly at the cell periphery – and growing faster. Together, these findings demonstrate that the interaction between talin and KANK1 bridges the CMSC to the adhesion sites, and controls the dynamics of microtubules throughout the cell.

However, several questions remain to be answered. Does the talin-KANK1 interaction play a role in regulating focal adhesion dynamics across a cell? Does this interaction control cell shape and migration? These and other unresolved questions should still be addressed in future studies. Without a doubt, elucidating how microtubules regulate cellular behavior via focal adhesions will advance our understanding of how cells work, and may also help to identify new therapeutic targets to suppress diseases such as cancer.
